# Implementation of Predictive Data Mining Techniques for Identifying Risk Factors of Early AVF Failure in Hemodialysis Patients

**DOI:** 10.1155/2013/830745

**Published:** 2013-06-04

**Authors:** Mohammad Rezapour, Morteza Khavanin Zadeh, Mohammad Mehdi Sepehri

**Affiliations:** ^1^Department of Industrial Engineering, Tarbiat Modares University, Tehran 14117-13114, Iran; ^2^Hasheminejad Clinical Research Development Center (HCRDC) and Rasoul Akram Hospital, Tehran University of Medical Sciences (TUMS), Iran; ^3^Hospital Management Research Center, Iran University of Medical Sciences, Tehran 19697-14713, Iran

## Abstract

Arteriovenous fistula (AVF) is an important vascular access for hemodialysis (HD) treatment but has 20–60% rate of early failure. Detecting association between patient's parameters and early AVF failure is important for reducing its prevalence and relevant costs. Also predicting incidence of this complication in new patients is a beneficial controlling procedure. Patient safety and preservation of early AVF failure is the ultimate goal. Our research society is Hasheminejad Kidney Center (HKC) of Tehran, which is one of Iran's largest renal hospitals. We analyzed data of 193 HD patients using supervised techniques of data mining approach. There were 137 male (70.98%) and 56 female (29.02%) patients introduced into this study. The average of age for all the patients was 53.87 ± 17.47 years. Twenty eight patients had smoked and the number of diabetic patients and nondiabetics was 87 and 106, respectively. A significant relationship was found between “diabetes mellitus,” “smoking,” and “hypertension” with early AVF failure in this study. We have found that these mentioned risk factors have important roles in outcome of vascular surgery, versus other parameters such as “age.” Then we predicted this complication in future AVF surgeries and evaluated our designed prediction methods with accuracy rates of 61.66%–75.13%.

## 1. Introduction

Chronic kidney disease (CKD) is a condition in which the kidneys are damaged and cannot filter blood as well as possible. In advanced stage of CKD known as end-stage renal disease (ESRD) kidney functions are reduced very severely. Hemodialysis (HD) treatment is the most common procedure which is performed for ESRD patients and HD requires permanent vascular access (VA) as an important aspect [1]. Furthermore, there are three main types of VA used in HD treatment: arteriovenous fistula (AVF), synthetic arteriovenous graft (AVG), and central venous catheter (CVC) [2]. Types of VA have their risks and can be expensive. According to recommendation of clinical practice guidelines, AVF is the access of first choice based on the reduced associated complications, morbidity, and mortality compared with AVG and CVC; also AVF has superior survival rate (estimated at 90% after one year) than other VA types (such as 60% of AVG) [3]. AVF is less expensive and remains the gold standard access to HD.

Since incidence of early AVF failure is reported as 20–60% [4], therefore detection of risk factors in early AVF failure is essential in caring for these HD patients in terms of medical, economic, and psychological impact. An AVF that is never usable for dialysis or that fails within three months of usage, should be classified as an early failure [5]. The goals of our study are to extract pattern of early AVF failure, predict this issue, and determine the high-risk factors on it, using data mining approaches.

 “Data mining” is defined as a step in the knowledge discovery in databases (KDD) process that consists of applying data analysis and discovery algorithms that, under acceptable computational efficiency limitations, produce a particular enumeration of patterns (or models) over the data [6]; also KDD is defined as the nontrivial process of identifying valid, novel, potentially useful, and ultimately understandable patterns in data. Data mining's approaches are boosting and their applications have become increasingly essential for healthcare organizations to make decisions based on the analysis of the huge amounts of clinical data generated by healthcare transactions. Data mining is becoming increasingly popular in healthcare, if not increasingly essential, and several factors motivated the use of data mining applications in healthcare, such as fraud and abuse detection, ability of transforming data, and benefit of healthcare providers [7]. Another factor is that data mining can improve decision making by discovering patterns and trends in large amounts of complex data [8]. Several studies employed data mining approaches to discover the knowledge of relation between the measured parameters and prevention of AVF failure. Temporal data mining techniques are studied [9] for dialysis failure prediction and analyzing the data of dialysis sessions coming from 43 different patients. *K*-means and expectation maximization algorithm are implemented [10] to cluster some attributes of HD patients. Data mining is considered in the medical settings of HD treatment and provided a brief review of state-of-the-art methods for predicting patient risk and survival of dialysis patients [11]. WJ.48 tree algorithm is used for analyzing data of 170 patients on dialysis for 12 or more months and interpreted patterns of high-risk groups in patients by extracting decision rules [12].

### 1.1. World Statistics

The prevalence of CKD is increasing around the world. In 2010, more than 10% of people or more than 20 million patients in the USA have CKD [13]. According to recent statistics of National Kidney Foundation (NKF), there are 26 million CKD patients in USA in 2012 [14]. Also the rate of ESRD as a chronic illness has grown rapidly in recent years [15]. At the end of 2004, ESRD is reported with a prevalent world population of 400,000, including over 300,000 hemodialysis (HD) patients [16]. At the end of 2008, 547,982 USA residents were under treatment of ESRD and 382,343 of them received dialysis, of which 354,443 were under hemodialysis [17].

In Iran more than 14,000 patients are treated with chronic HD therapy for ESRD [18]. AVF is used by 93.4% of Iranian HD patients [1]. This finding exceeds the recommendations by guidelines and the percentage of 67–91% reported by AVF usage in many Western countries in recent years [19].

## 2. Materials and Methods

### 2.1. Patient Population

This study comprises AVF data of 193 patients who were under hemodialysis (HD) in Hasheminejad Kidney Center (HKC) of Tehran, which is one of Iran's largest renal hospitals. There were 137 male (70.98%) and 56 female (29.02%) patients introduced into this study. The average of age for all the patients was 53.87 ± 17.47 years. Twenty eight patients had smoked and the number of diabetic patients and nondiabetics was 87 and 106, respectively. In previous study, we clustered the attributes of early AVF failure, using descriptive approaches with *n* = 99 patients [20].

In present study, we have two datasets of this vascular surgery: one of datasets was collected during period from year 2005 to 2006 and contains 36 parameters of 99 patients who all had early AVF failure; second dataset was collected from December to November 2010 and contains 25 parameters of 94 patients of whom 87 patients had survival surgery and the remaining (7 patients) had early AVF failure. So, by merging these two datasets we have 106 patients with early AVF failures and 87 patients without AVF failure.

### 2.2. Data Parameters

We merged two datasets and found eight similar parameters of them, where each patient is characterized by seven attributes: age (age of patients), sex (male or female), htn (hypertension), DiabetesM (diabetes mellitus), Hgb (hemoglobin), smoking (yes or no), and location (site of AVF with brachial or radial). The last column thrombosis (yes: failure or no: survival) is the designated *class* attribute. So we obtained a final dataset with 8 parameters of 193 records (patients), which contain 106 cases of failures and 87 cases of survivals.

### 2.3. Methodologies

In present study, we use supervised techniques of data mining to predict early AVF failure and determine the risk factors that have important roles on it. Many numbers of possible choices in Weka operators are available; we performed many types of them and consulted the surgeon about obtained decision trees (DT). Our choosing of the operator was on the bases that its extracted DT contains new knowledge for help in next vascular surgeries and finally due to the best accuracy rate between prediction methods (as we will see in part of “validation of analyses”). Sampling data for predictions (and number of training patients) are selected randomly. We present a brief description of the learner algorithms used in this work: JRIP and J48.

JRIP rule learner was proposed by Cohen as an optimized version of learning algorithm IREP (incremental reduced error pruning); JRIP implements repeated incremental pruning to produce error reduction (RIPPER) in Java, a prepositional rule learner [21]. Rules are created for every class in the training set and are then pruned. In this algorithm, the discovered knowledge is represented in the form of IF-THEN prediction rules, which have the advantage of being a high-level and symbolic knowledge representation contributing towards the comprehensibility of the discovered knowledge [22]. The method is based on the construction of a rule set in which all positive examples are covered. Initially, the current set of training examples are partitioned into two subsets, a growing set and a pruning set. The rule is constructed from examples in the growing set. The rule set initiates with an empty rule set and rules are added incrementally to the rule set until no negative examples are covered. After that, JRIP replaces or revises individual rules by using reduced error pruning in order to increase the accuracy of rules. It replaces or revises individual rules by using reduced error pruning. To prune a rule the algorithm takes in account only a final sequence of conditions from the rule and sorts the deletion that maximizes the function [23].

J48 decision tree is an implementation of the well-known Quinlan algorithm (C4.5) [24], which is an improvement algorithm, derived from basic ID3 induction system using the standard TDIDT (top-down induction of decision trees) approach, recursively partitioning the data into smaller subsets based on the value of an attribute [24, 25]. The “pruned” version of J48 reduces the chances of overfitting the data [26]. This classifier builds a decision tree for the given dataset, whose nodes represent discrimination rules acting on selective features by recursive partitioning of data, using depth-first strategy. The algorithm uses the fact that each attribute of the data can be used to make a decision by splitting the data into smaller subsets. To make the decision, the algorithm considers all the possible tests that can split the dataset and culls a test that gives the highest information gain. For each discrete attribute, one test with outcomes for each distinct value of the attribute is considered. For each continuous attribute, binary tests involving every distinct value of the attribute are considered. In order to gather the entropy gain of all these binary tests efficiently, the training data set belonging to the node in consideration is sorted for the values of the continuous attribute and the entropy gains of the binary cut based on each distinct values are calculated in one scan of the sorted data. Then a new feature is chosen and the splitting process is repeated for each attribute in a recursive manner until further splitting is not gainful. In the resulting tree structure; each inner node in the tree corresponds to an attribute, each branch represents a possible value or range of values of that attribute and each leaf represents the predicted value of target attribute [23].

The numbers in (parentheses) at the end of each leaf tell us the number of examples in this leaf. If one or more leaves were not pure (= all of the same class), the number of misclassified examples would also be given, after a slash (“/”) [27].

#### 2.3.1. Rule Mining

At first, we analyze on seven parameters, without considering side of AVF in data (location attribute) in this stage. We extract the rules embedded in data, using “JRIP” operator. This classifier implements a propositional rule learner from data. The obtained rules in Table 1 show a determining role of diabetes mellitus (DM) in AVF failure.

**Figure 1:**
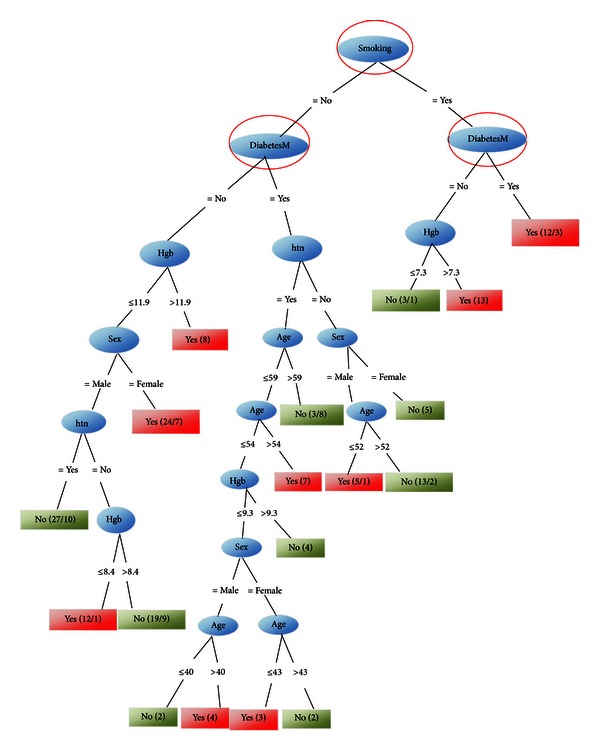
Effects of diabetes and smoking are determined from interpreted rules of this tree.

The extracted tree after running J48 over the data shows smoking and DM as risk factors (Figure 1).

The second operator is “rule learner” and its results are shown in Table 2.

If we consider the parameter “location” of AVF in our analysis, the extracted rules are shown in Table 3. Also from Tables 2 and 3 it seems that “htn” (hypertension) is a very important parameter.

**Figure 2:**
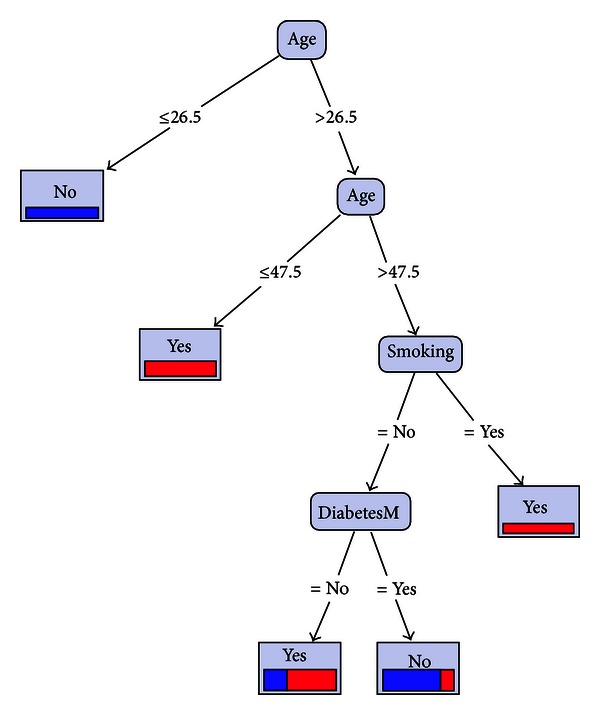
Decision tree of system training, after stratified sampling.

*Prediction I.* Now we select training data, from original data by “stratified sampling” method. For this purpose, we choose sample_ratio = 0.1; then 20 samples are selected, where 11 numbers of them were in class “yes” and 9 numbers in class “no.” The rules that are hidden in this part of data will be detected after system training using decision tree, as shown in Figure 2.

Therefore in above selected data, three parameters have important roles: age, smoking, and diabetes. Now we predict the AVF failure here as the first method, as shown in Table 4. So, the new field “prediction (failure)” will be created. 

**Figure 3:**
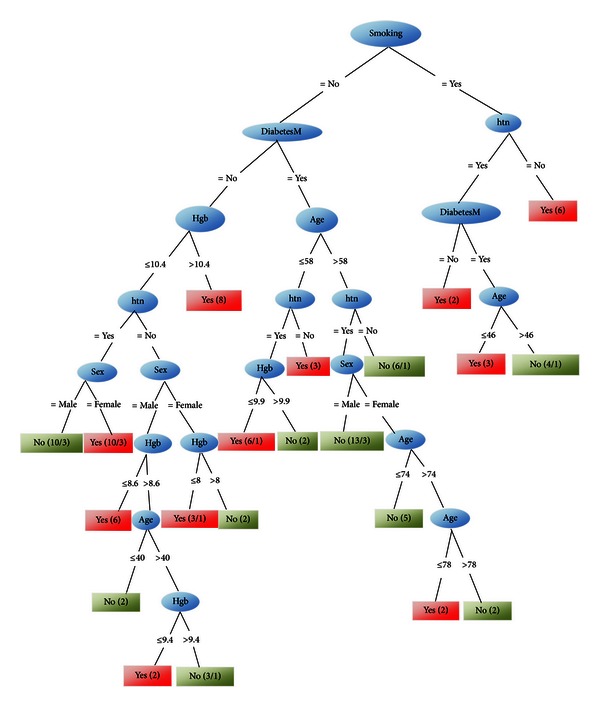
System training by J48 tree, after absolute stratified sampling.

*Prediction II.* In the second method by “absolute stratified sampling” and system training with J48 tree, we finalize the tree of Figure 3.

Thus the AVF failure prediction is due to Table 5.

Comparing the predicted results in Tables 4 and 5 shows that the results of method II are nearer to the real events. We will see this fact in assessment section exactly; also here we denote that in corresponding figures with recent tables, Figure 3 is more compatible with medical experiences of the surgeon. Hence we prefer Figure 3 over Figure 2 and follow that role of “smoking” and “diabetes” (in Figure 3) is more effective than that of “age” (in Figure 2) as high-risk factors in early AVF failure.

## 3. Results

Existence of either diabetes mellitus or smoking in HD patients increases early AVF failure in their surgery. We designed two applied methods and at first predicted risk factors of this complication with accuracy rates of 61.66%–74.61%. Then we added data of side of AVF (location in hand) to the data and predicted this complication with accuracy rates between 67.91% and 75.13%. Results support the impressive roles of risk factors in AVF failures. We found that “diabetes mellitus,” “smoking,” and “hypertension” have important roles in early AVF failure, which are more effective roles than other factors such as “age.”

### 3.1. Approaches Assessment

In our study, as described previously, the separate effects of diabetes, smoking, and hypertension, respectively, are determined in accordance with the extracted rules (in Tables 1–3). Also the role of them together can be found in Figures 1–3. As we described earlier, we designed two methods of sampling and system training and analyzed them. Furthermore we measured the accuracy rates of these methods and saw their rates between 61.66% and 74.61% (Tables 6 and 7).

In the first method (“stratified sampling” and system training with decision tree), we have the accuracy rate equal to 61.66% (Table 6).

Also the accuracy rate of method 2 will measure equal to 74.61% (Table 7).

Therefore due to accuracy rates, the predicted results in the 2nd method are better approximated than those of the 1st method. Therefore we denote that Diabetes and then smoking are more effective than age in AVF failure (i.e., higher factor in tree of method 1).

Finally, we added the surgery side parameter (location) and also we surveyed two other methods using “neural network” and “naïve Bayesian.” Then we predicted early AVF failure with better accuracy rates of 67.91%–75.13% which says an important role of the “location.” So we obtained the numbers of “predicted patients with/without early AVF failure” and rate of any recent methods in Table 8.

To compare above results with real end points, remember that we had 193 patients with 106 cases of failure and 87 cases of survival of AVF.

## 4. Discussion and Conclusions

Extracting potential knowledge from vascular surgery information is showed using data mining techniques. In the proposed analysis of early AVF fistula phenomenon we can find vulnerable patients and determine decision about using AVF or replace it by other methods as AVG. Ultimate benefits are diagnosed and control risk factors of patients or making recommendations for them. 

In present study we detected the high-risk factors and predicted the risk of AVF early failure in patients, using supervised techniques. These approaches give a better functionality to expert system of hospitals. Scientific prediction and control of the AVF failure can help achieving the target of percent AVF use in the prevalent HD population. Additionally, the prediction of AVF failure has a major role in the planning in these cases: if the percentage of failure was high, they may use other surgery methods such as the AVG; if the risk of fistula failure was low, they may use the same method (AVF) but make recommendations to the patient and his/her family, such as at what level they should keep the blood pressure of the patient after surgery.

We examined the risk factors of early AVF failure in vascular access surgery. The research was studied in a real society of hemodialysis (HD) patients and we concluded that diabetes, smoking, and hypertension then are risk factors of early AVF failure. We used data mining techniques and designed prediction approaches to predict probability of this complication in new HD patients whom have been referred by nephrologists to AVF surgery.

In conclusion, these outcomes enable early detection of those patients that have a higher risk and can guide the surgeon to select the surgical procedure. Moreover, our additional clinical sequent is that proposes the decision about selecting more useful vascular access method to guide the surgeon and improve patient safety. Finally, we demonstrate the necessity of using data mining techniques to discover clinically relevant knowledge.

## Figures and Tables

**Table 1:** The extracted rules with JRIP learner (“yes” means early AVF failure).

	JRIP rules
	(DiabetesM = yes) → Failure = no (87.0/37.0) → Failure = yes (106.0/37.0)
	Number of rules: 2

**Table 2:** The extracted rules by running “rule learner.”

	Rule model
	If DiabetesM = no and sex = female, then yes (7/19)
	If DiabetesM = no and htn = no, then yes (13/28)
	If htn = no and age = range2 [49.50–64.50], then no (10/1)
	If sex = male and Hgb = range2 [8.45–9.95], then yes (7/13)
	If age = range3 [64.50–*∞*] and DiabetesM = yes, then no (18/10)
	If Hgb = range3 [9.95–*∞*] and sex = male, then yes (8/16)
	If Hgb = range3 [9.950–*∞*], then no (4/0)
	If sex = male and age = range3 [64.500–*∞*], then no (4/2)
	If sex = female and age = range1 [−*∞*–49.5], then yes (2/4)
	If Hgb = range1 [−*∞*–8.45] and DiabetesM = yes, then no (8/4)
	If sex = female and htn = yes, then yes (1/4)
	If age = range2 [49.5–64.5] and sex = male, then no (3/1)
	If sex = male, then yes (2/4)
	Correct: 135 out of 193 training examples.

**Table 3:** Extracted rules, analysis with side of AVF.

	Rule model
	If location = brachial and DM = no, then yes (30/2)
	If location = radial and DM = yes, then no (24/45)
	If dm = yes and Hgb = range2 [8.450–9.950], then yes (5/0)
	If Hgb = range3 [9.950–*∞*] and htn = yes, then yes (11/5)
	If age = range1 [−*∞*–49.500] and Hgb = range3 [9.950–*∞*], then yes (7/1)
	If age = range2 [49.50–64.50] and sex = female, then no (0/3)
	If Hgb = range3 [9.950–*∞*], then no (0/4)
	If htn = no and Hgb = range1 [−*∞*–8.450], then yes (9/4)
	If age = range2 [49.500–64.500] and DM = no, then no (1/5)
	If htn = yes and sex = female, then yes (4/2)
	If sex = female and htn = no, then no (1/2)
	If age = range3 [64.500–*∞*] and Hgb = range1 [−*∞*–8.450], then no (2/5)
	If htn = yes and age = range3 [64.500–*∞*], then yes (2/0)
	If Hgb = range1 [−*∞*–8.450] and DM = yes, then yes (1/0)
	If Hgb = range2 [8.450–9.950] and htn = yes, then no (1/2)
	If htn = yes and sex = male, then yes (3/2)
	If sex = male, then no (5/5)
	Correct: 143 out of 193 training examples

**Table 4:** The prediction results by method one.

Id	Integer	Avg = 97 ± 55.714	[1.0; 193.0]
Failure	Nominal	Mode = yes (106), least = no (87)	No (87), yes (106)
Prediction (failure)	Nominal	Mode = yes (112), least = no (81)	No (81), yes (112)
Confidence (no)	Real	Avg = 0.457 ± 0.380	[0.0; 1.0]
Confidence (yes)	Real	Avg = 0.543 ± 0.380	[0.0; 1.0]

**Table 5:** The prediction results by the 2nd method.

Id	Integer	Avg = 97 ± 55.714	[1.0; 193.0]
Failure	Nominal	Mode = yes (106), least = no (87)	No (87), yes (106)
Prediction (failure)	Nominal	Mode = yes (105), least = no (88)	No (88), yes (105)
Confidence (no)	Real	Avg = 0.408 ± 0.371	[0.0; 1.0]
Confidence (yes)	Real	Avg = 0.592 ± 0.371	[0.0; 1.0]

**Table 6:** The accuracy rate in method one.

Accuracy: 61.66%			
	True no	True yes	Class precision
Pred. no	47	34	58.02%
Pred. yes	40	72	64.29%
Class recall	54.02%	67.92%	

**Table 7:** The accuracy rate in method two.

Accuracy: 74.61%			
	True no	True yes	Class precision
Pred. no	63	25	71.59%
Pred. yes	24	81	77.14%
Class recall	72.41%	76.42%	

**Table 8:** Final predicted results in analysis with *location*.

Sampling method	Training algorithm	Prediction results	
Stratified	Neural network	Accuracy:	69.97% (±5.17%)
		Yes/no:	74/119
Stratified	Decision tree	Accuracy:	67.91%
		Yes/no:	94/99
Stratified	Naïve Bayesian	Accuracy:	69.97% (±8.61%)
		Yes/no:	100/93
Absolute stratified	WJ-48	Accuracy:	75.13%
		Yes/no:	96/97

## Abbreviations

AVF: Arteriovenous fistula
AVG: Arteriovenous graft
CDC: Centers for Disease Control and Prevention
CKD: Chronic kidney disease
CVC: Central venous catheter
DM: Diabetes mellitus
DT: Decision tree
ESRD: End-stage renal disease
HD: Hemodialysis
HKC: Hasheminejad Kidney Center
IREP: Incremental reduced error pruning
KDD: Knowledge discovery in databases
MS: Master of Science
NKF: National Kidney Foundation
RIPPER: Repeated incremental pruning to produce error reduction
TDIDT: Top-down induction of decision trees
TMU: Tarbiat Modares University
TUMS: Tehran University of Medical Sciences
USRDS: United States Renal Data System
VA: Vascular access.
